# Emerging Herbal Cosmetic Production in Sri Lanka: Identifying Possible Interventions for the Development of the Herbal Cosmetic Industry

**DOI:** 10.1155/2021/6662404

**Published:** 2021-03-10

**Authors:** Dehel Gamage Nadeeshani Dilhara Gamage, Rathnayaka Mudiyanselage Dharmadasa, Don Chandana Abeysinghe, Rathnayaka Gamlathge Saman Wijesekara, Gamika A. Prathapasinghe, Takao Someya

**Affiliations:** ^1^Faculty of Agriculture and Plantation Management, Wayamba University of Sri Lanka, Makandura, Gonawila 60170, Sri Lanka; ^2^Industrial Technology Institute, 363, Bauddhaloka Mawatha, Colombo 7, Sri Lanka; ^3^Faculty of Livestock, Fisheries and Nutrition, Wayamba University of Sri Lanka, Makandura, Gonawila 60170, Sri Lanka; ^4^ALBION Co., Ltd., Ginza 1-7-10, Chuo-ku, Tokyo 104-0061, Japan

## Abstract

Although the herbal cosmetic industry has exponentially increased globally, manufacturing of herbal cosmetic products in Sri Lanka is still very limited. Therefore, objectives of the study were to recognize plants used in commercialized herbal cosmetic products and major constraints faced by herbal cosmetic manufacturers and to identify possible interventions for the development of herbal cosmetic industry of Sri Lanka. Information was gathered via a semistructured questionnaire by personal interviews with 11 large-scale multiple herbal cosmetic products manufacturers. Collected data were analyzed via frequency index for usage and descriptive statistics. A total of 115 plant species belonging to 56 families were identified. Extensive usage of *Aloe vera* (L.) Burm.f., *Coscinium fenestratum* (Goetgh.) Colebr., and *Santalum album* L. (90.91%) was reported among herbal cosmetic manufacturers. The highest number of plants or plant materials was used for manufacturing skin care products (54.78%) followed by hair care (19.13%) and oral care (6.96%). The majority of plants were reported from the plant family Fabaceae (16 species). Leaves (20.87%) were the widely used plant part, whereas 10 plant species were used as whole plants. Inadequacy of 7 plants/plant materials and importation of 8 plant materials for the production were also recognized. As major constraints faced by herbal cosmetic manufacturers, inadequate availability and poor quality of raw materials were emphasized. In conclusion, establishing proper cultivation system, implementing strategies for quality control of raw materials, and conducting ethnobotany, ethnopharmacological surveys to identify cosmetic potential of medicinal plants and partnerships with universities to transfer technology for product development to industries are possible interventions for the development of herbal cosmetic industry of Sri Lanka.

## 1. Introduction

Sri Lanka, formerly known as Ceylon, is an island with an area of approximately 65,610 km^2^. Despite its relatively small size, Sri Lanka possesses a high level of biodiversity due to its varied climate and topographical conditions [1]. Plants have been used for treating illnesses over a thousand years by four systems of traditional medicine in Sri Lanka called *Ayurveda*, *Siddha*, *Unani*, and *Deshiya chikitsa* [2]. As a biologically diverse country in Asia, Sri Lanka currently possesses 29.7% of forest cover [3], 4143 plant species distributed within 214 families, and 1522 genera. Of these, 1025 plant species are endemic to the country [4]. Among the native flora of Sri Lanka, more than 1,400 plants are used in indigenous medicine [5]. Many formulae for medicinal preparations of Sri Lankan traditional system of medicine are handed down from generation to generation or are found only in the scripts of old “ola leaf” books treasured by traditional and Ayurvedic practitioners [6]. The study conducted by Kankanamalage et al. [7] reveals the sources of medicinal plant materials that are obtained for numerous medicinal plant-based trades. About 71.13% of these medicinal plants/herbal materials are obtained from local sources, 26% are imported, while 2.87% are obtained through either route. Moreover, it reveals that 80% of both fresh and dry plant materials contribute to the herbal industry. Thus, it clearly implies the importance of medicinal plants in different systems of medicine in Sri Lanka. Moreover, the study conducted by Dissanayake [8] on “Medicinal plant research in Sri Lanka: a scientometric study based on Scopus database” highlights about research studies of 190 plants including 22 endemic plants. It reveals that most of the conducted studies were activity-based studies such as toxicity, antibacterial, antifungal, hypoglycemic, antioxidant, anti-inflammatory, and diuretic. This was followed by general studies such as physicochemical, chemical, postharvest, horticultural, and propagation studies of plants. This study evidently indicates the largely unexplored knowledge gap of medicinal plants in Sri Lanka.

The global consumption of herbal cosmetics has shown spectacular growth in recent years due to the growing recognition of long-term health benefits [9, 10]. According to the available market research, the current global natural and organic personal care products market is valued approximately US$ 11 billion and is expected to reach US$ 22 billion by 2022 [11]. Thus, the global enormous demand for herbal cosmetics results in a huge trade from local to the international level. Consequently, there are ample opportunities for Sri Lanka to expand its herbal cosmetic productions through its unique biodiversity of medicinal flora and a rich base of traditional knowledge. However, there has been a dearth of published information on herbal cosmetic production and plants used in the herbal cosmetic industry of Sri Lanka. Although the herbal cosmetic industry has significantly increased throughout the world, the supply of herbal cosmetic products from Sri Lanka is still very limited. Therefore, it is crucial to identify major constraints faced by herbal cosmetic manufacturers to identify possible interventions for the development of the herbal cosmetic industry of Sri Lanka. At present, this becomes a necessary area to be addressed within the country as this is one of industries which can easily capitalize the global trends. Thus, the objective of this present survey was to identify plants and plant parts used in commercialized herbal cosmetic products in Sri Lanka especially under the categories of skin care, hair care, and oral are. The constraints faced by herbal cosmetics manufacturers and their suggestions were also studied to identify possible interventions for the future improvements of the herbal cosmetic industry in Sri Lanka.

## 2. Materials and Methods

### 2.1. Study Area and Selection of Respondents

Any herbal product to be sold to the public must be registered under the Department of Ayurveda, Sri Lanka. Therefore, herbal cosmetic manufacturers were selected based on the registry maintained for herbal products at the Department of Ayurveda, Sri Lanka. This survey was conducted from January to August 2018. In total, 18 herbal cosmetic manufacturers in Sri Lanka were identified. Among these, 13 herbal cosmetic manufacturers were pioneers in the herbal cosmetic industry of Sri Lanka, while the rest were small-scale herbal cosmetic manufacturers. Prior to data collection, each respondent was informed of the aims and objectives of the study to obtain their consent and cooperation for the survey. However, permission was received only from 11 large-scale herbal cosmetic manufacturers to carry out the survey and to gather the information. Among them, 9 manufacturing factories were in the Western Province, Sri Lanka, while other two factories were in the Central Province and Southern Province, Sri Lanka, respectively.

### 2.2. Preparation of Questionnaire

Information was collected via a semistructured questionnaire (Supplementary 1) under two main sections. The first section was designed to gather general information about the organization including the organization name/name of the organization representative, experience of the profession, address, province/district, and registration number assigned by Ayurveda Department. The second section of the questionnaire was prepared to gather the following major information: (1) manufacturing herbal cosmetic products under skin care, hair care, and oral care categories; (2) medicinal plants and plant parts used for productions; (3) sources (local/import) of herbal materials; and(4) availability (sufficient/insufficient) of herbal materials. Furthermore, main challenges and difficulties in herbal cosmetics production and suggestions were recorded for identifying future improvements of the herbal cosmetic industry in Sri Lanka.

### 2.3. Data Collection and Quantitative Analysis

Personal interviews were conducted. During the survey, the information mentioned in Section 2.2 was specifically obtained. Plants/plant materials were collected at the manufacturing sites. Furthermore, collected plants/plant materials were dried, preserved, and mounted on herbarium sheets. Herbarium voucher numbers were coded from NGHC 01 to NGHC 115 as the same order of the plant list indicated in Table 1. Plant identification was carried out by comparing the deposited herbarium of the royal botanical garden, Peradeniya, Sri Lanka. The scientific names of plants were validated based on the collections listed in the homepage (http://www.theplantlist.org). Vernacular names and English names of plants were verified using the Ayurveda authentic books, “Compendium of medicinal plants, Sri Lankan study,” volumes I to IV issued by Ayurveda Department of Sri Lanka and “A collection of medicinal plants in Sri Lanka,” issued by Nature's Beauty creations limited, Sri Lanka. Collected data were tabulated and analyzed using descriptive statistics. The frequency index for usage of each plant was calculated using the following formula described by Dharmadasa et al. [12]:

(1) $$\begin{array}{c} \text{Frequency Index}=\frac{n}{N}\times 100, \end{array}$$

where “*n*” is the total number of cosmetic manufacturers who listed a particular plant species for their cosmetic productions and “*N*” is the total number of large-scale multiple range of cosmetic products manufacturers.

## 3. Results and Discussion

### 3.1. General Overview about Herbal Cosmetic Manufacturers in Sri Lanka

**Figure 1:**
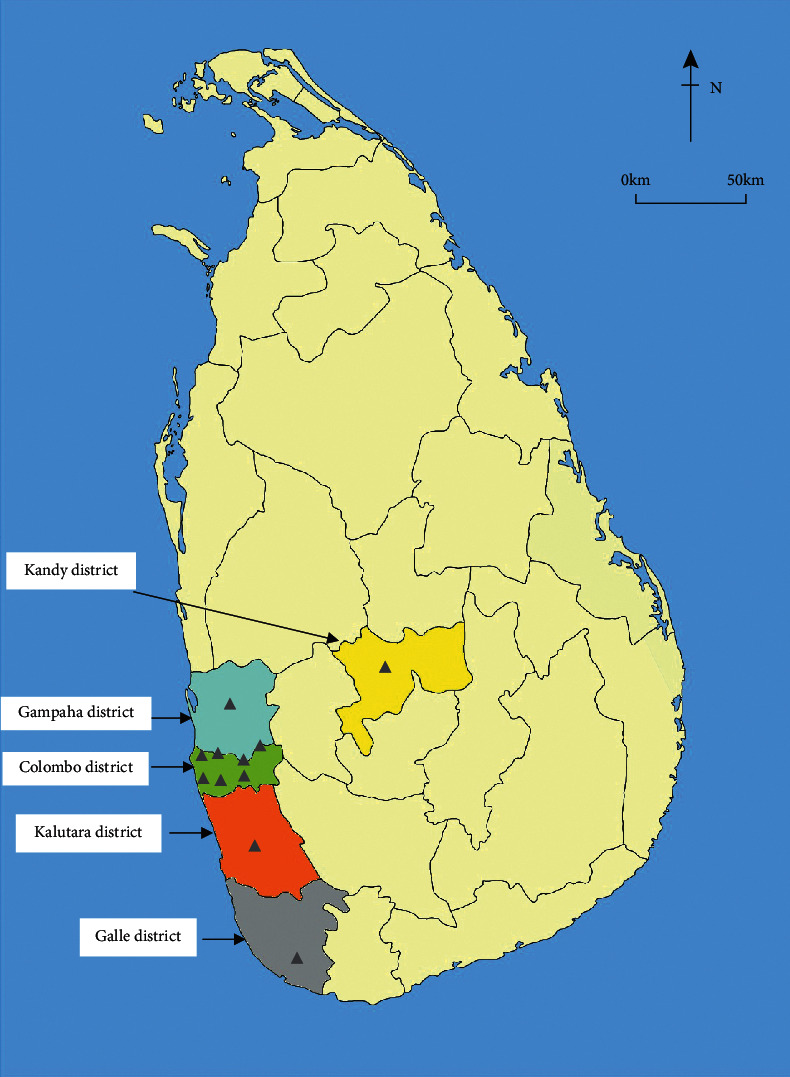
Locations of large-scale herbal cosmetic manufacturers in Sri Lanka.

The total respondent percentage was 61 from requested herbal cosmetic manufacturers. All, 13 large-scale manufacturers produce multiple products under the categories of skin care, hair care, and oral care, whereas small-scale manufacturers produce only single products. 7 herbal cosmetic manufacturers refused to provide necessary information to support the survey because their formulae were based on family recipes. However, 10 leading large-scale private sector herbal cosmetic manufacturers and a state herbal cosmetic manufacturer cum under the Ministry of Indigenous medicine, Sri Lanka, were interviewed to gather the information. Totally, 9 cosmetic manufacturers were located in Colombo, Kalutara, and Gampaha districts belonging to Western Province, while other 2 are located in Galle District of Southern Province and Kandy District of Central Province, respectively (Figure 1). Of these, 5 companies only cater for the local market, whereas 6 companies cater for both local and international markets. This is further proven by the list of Ayurvedic herbal products exporters in Sri Lanka published on the website of Export Development board, Sri Lanka [13]. Furthermore, multiple herbal cosmetic products under skin care, hair care, and oral care categories are manufactured by all interviewed herbal cosmetic manufacturers. Creams (moisturizer, fairness creams, night creams, and foot creams), lotions (body lotions), soap, cleansers (body wash, face wash, and hair shampoo), gel (hair, body, and face), scrubs (face, body, and foot), and mask (powder pack, creamy face masks, and foot pack) are some of main products manufactured under the skin care and hair care category, while herbal toothpastes and mouth washes are major products manufactured under the oral care category.

### 3.2. Plant Families

A total of 115 different plant species belonging to 56 families were documented. The most dominant family was reported as family Fabaceae (13.91%, 16 species). This was followed by Rutaceae (6.09%, 7 species), Asteraceae (5.22%, 6 species), Poaceae, Zingiberaceae, Malvaceae (4.35%, 5 species per each), Moraceae, Myrtaceae (3.48%, 4 species per each), Rosaceae, Rubiaceae (2.61%, 3 species per each), Apiaceae, Apocynaceae, Arecaceae, Combretaceae, Euphorbiaceae, Lamiaceae, Lauraceae, Lythraceae, Menispermaceae, Oleaceae, Sapindaceae (1.74%, 2 species per each), and one species each for the rest of families. The greatest utilization of medicinal plants in family Fabaceae under different disciplines such as Ayurveda, traditional systems of medicine, and medicinal plant-related industries in Sri Lanka has also been reported in [2, 7, 12, 14]. Furthermore, these studies highlight the prominent usage of medicinal plants in Rutaceae, Asteraceae, Poaceae, Zingiberaceae, Malvaceae, Apocynaceae, Euphorbiaceae, and Lamiaceae plant families.

### 3.3. Plants Used in Different Cosmetic Products

**Figure 2:**
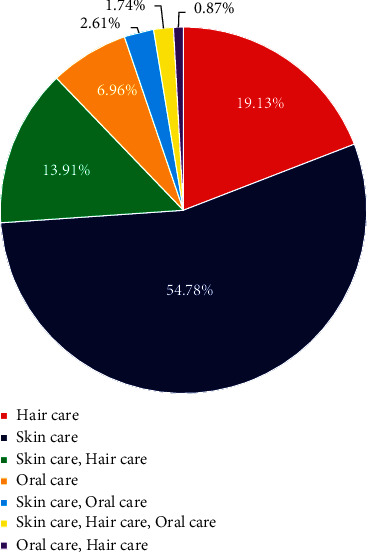
Plants usage in different cosmetic product categories.

As indicated in Figure 2, plant species used for cosmetic purposes can be classified into three main categories called skin care, hair care, and oral care. Out of 115 plants, the highest number of plants/plant materials was used in skin care products. It was reported as 63 plant species (54.78%). 22 plant species (19.13%) were used for hair care products, while 8 plant species (6.96%) were used in oral care products. However, 16 plant materials (13.91%) were used in both skin and hair care products, whereas 3 plant materials (2.61%) were used in both skin and oral care products. Furthermore, usage of *Terminalia chebula* Retz in both oral and hair care products as well as usage of both *Glycyrrhiza glabra* L. and *Cinnamomum verum* J.Presl in all three cosmetic products categories were also reported. Moreover, research study conducted by Nirmalan [14] on cosmetic perspectives of ethnobotany in Northern part of Sri Lanka confirms the higher usage of local plants for skin care followed by hair care.

### 3.4. Widely Used Plants for Herbal Cosmetic Preparations

High-frequency usage of *Aloe vera* (L.) Burm.f., *Santalum album* L*.,* and *Coscinium fenestratum* (Goetgh.) Colebr. among 10 cosmetics manufacturers out of eleven was reported. Frequency indexes of these plants were reported as 90.91%. *Azadirachta indica* A.Juss. and *Kokoona zeylanica Thwaites* showed the second highest usage followed by *Phyllanthus emblica* L. Frequency indexes of *Azadirachta indica* A.Juss. and *Kokoona zeylanica Thwaites* were reported as 72.73% and 63.64% for *Phyllanthus emblica* L. Moreover, *Citrus aurantiifolia* (Christm.) Swingle, *Centella asiatica* (L.) Urb., *Curcuma longa* L., *Caryophyllus aromaticus* L., *Curcuma aromatica* Salisb., *Eclipta prostrata* (L.) L., *Indigofera tinctoria* L., and *Glycyrrhiza glabra* L. showed 54.55% usage among cosmetic manufacturers. Furthermore, ethnobotanical surveys conducted to find out medicinal plants and plant parts used in skin diseases [6], snake bite treatments [12], and anti-inflammatory remedies [2] in Sri Lanka revealed about greater usage of *Azadirachta indica* A.Juss. and *Curcuma longa* L. in skin disease treatments, *Citrus aurantiifolia* (Christm.) Swingle in snake bite treatments, and *Coscinium fenestratum* (Goetgh.) Colebr. in anti-inflammatory remedies, respectively. According to the survey on medicinal materials used in traditional systems of medicine in Sri Lanka, *Centella asiatica* (L.) Urb., *Phyllanthus emblica* L., *Azadirachta indica* A.Juss., and *Indigofera tinctoria* L. have been listed under the most demanded medicinal plants in Sri Lanka by Kankanamalage et al. [7].

### 3.5. Different Plant Parts Used in Cosmetic Productions

**Figure 3:**
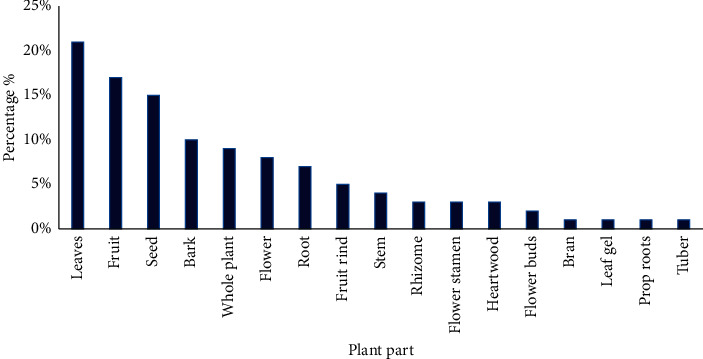
Different parts of the plants used in herbal cosmetics manufacturing of Sri Lanka.

As shown in Figure 3, a wide range of plant parts are used for herbal cosmetics manufacturing in Sri Lanka. Identified plant parts were reported as leaves, fruit, seeds, bark, flower, root, stem, fruit rind, rhizome, flower stamen, heartwood, flower buds, bran, leaf gel, prop roots, and tuber. The most utilized plant part was reported as leaves (20.87%). This was followed by fruit (17.39%), seed (14.78%), bark (9.57%), flower (7.83%), root (6.96%), fruit rind (5.22%), stem (4.35%), rhizome (3.48%), flower stamen, heartwood (2.61% per each), flower bud (1.74%), bran, leaf gel, prop roots, and tuber (0.87% per each), respectively. Usage of whole plant was recorded as 8.70%. In line with the studies of Napagoda et al. [2], Kumarasinghe [6], Kankanamalage et al. [7], Dharmadasa et al. [12], and Nirmalan [14], leaves were the commonest part of plants used in different treatments of Ayurveda, traditional system of medicine, and medicinal plant-based industries in Sri Lanka. Moreover, Kumarasinghe [6] stated that availability of most effective ingredients in leaves results in the highest usage. However, more scientific studies related to this area are required. As other reasons, Dharmadasa et al. [12] highlights availability in large quantities, easy accessibility, and cheaper costs for the abundance usage of leaves. These are in further agreement with scientific studies conducted by Miraldi et al. [15], Ghorbani [16], and Mowobi et al. [17] from other countries.

### 3.6. Availability of Plant Materials for Cosmetic Productions

Most of the cosmetic manufacturers mentioned about inadequate availability of 7 plant materials out of 115 plants for cosmetic productions in line with the existing demand. As they stated, *Mesua ferrea* L., *Kokoona zeylanica Thwaites, Dillenia retusa* Thunb., *Coscinium fenestratum* (Goetgh.) Colebr.*, Melaleuca leucadendra* (L.) L.*, Santalum album* L., and *Bacopa monnieri* (L.) Wettst. plant materials belonging to following plant families Calophyllaceae, Celastraceae, Dilleniaceae, Menispermaceae, Myrtaceae, Santalaceae, and Plantaginaceae were insufficient. Of these, *Kokoona zeylanica Thwaites* and *Dillenia retusa* Thunb are endemic to Sri Lanka. According to the viewpoint of cosmetics manufacturers, the main motive for the scarcity of these plant materials is lack of proper cultivation system within the country. Furthermore, Kankanamalage et al. [7] has revealed that *Coscinium fenestratum* (Goetgh.) Colebr. and *Bacopa monnieri* (L.) Wettst. are heavily used plants in Sri Lanka. Likewise, both Kankanamalage et al. [7] and Dharmadasa et al. [12] have mentioned about the limited availability of *Santalum album* L. in the Sri Lankan market.

### 3.7. Sources of Plant Materials for Cosmetic Productions

As cosmetics manufacturers stated, most of plants or plant materials used for cosmetics productions are bought from either local growers or local suppliers. However, *Pterocarpus santalinus* L.f., *Glycyrrhiza glabra* L., *Olea europaea* L.*, Cedrus deodara* (Roxb. ex D.Don) G.Don, *Prunus armeniaca* L.*, Fragaria* ×  *ananassa* Duchesne, *Santalum album* L., and *Withania somnifera* (L.) Dunal belonging to plant families Fabaceae, Oleaceae, Pinaceae, Rosaceae, Santalaceae, and Solanaceae were reported as importing plant materials for cosmetic productions. These results are further confirmed according to the survey conducted by Kankanamalage et al. [7].

### 3.8. Difficulties Encountered during the Collection of Plant Materials for Cosmetic Productions

Majority of cosmetics manufacturers emphasized about several constraints that they faced during the collection of plant materials for cosmetic productions. As they highlighted, quality of plant materials was one of the major constraints. Most of cosmetic manufacturers believed that imported plant materials are adulterated, or the active ingredients are partially extracted which cannot be identified through physical observations during buying. Furthermore, harvesting restrictions imposed by the government for some wild species such as *Coscinium fenestratum* (Goetgh.) Colebr which owns the highest frequency usage in the industry has created a greater impact on continuous productions due to declining suppliers. On the contrary, insufficient or absence of continuous supply of plants/plant materials was another major issue faced by cosmetic manufactures. For example, lack of growers and suppliers of *Bacopa monnieri* (L.) Wettst. has become a big issue as a large quantity is required in fresh state for the production. All statements given by cosmetic manufactures were clearly evident for the lack of proper cultivation system within the country. Moreover, most of cosmetic manufacturers revealed about sales issues of locally manufactured herbal cosmetic products as foreign cosmetic products have currently invaded the cosmetic market in Sri Lanka.

### 3.9. Knowledge Gaps and Directions for Future Researches

As Napagoda et al. [18] stated, only handful of scientific evidence are available on bioactivity studies of medicinal plants in Sri Lanka that could lead to the development of herbal cosmetics. Apart from the study on “Cosmetic perspective of ethnobotany in Northern part of Sri Lanka” [14], there has been hardly any ethnobotany report on cosmetic potential of medicinal plants in Sri Lanka. Therefore, ethnobotanical and ethnopharmacological surveys to identify the cosmetic potential of medicinal plants are crucial because most of traditional knowledge on medicinal plants and treatment are passed from generation to generation within families in Sri Lanka. Furthermore, establishing proper systematic cultivation systems for identified medicinal plants is paramount to overcome the insufficient and absence of continuous supply of raw materials for the production. This field must cover the preparation of growing media, preparation of seed or seedling, preparation of land, and preparation of seedbed, fertilizing, harvesting, and postharvest practices. Utilization of biotechnology such as tissue culture will benefit to preserve biodiversity through utilizing plants which are endangered or unavailable through conventional production or wild crafting. Moreover, conducting efficacy/safety tests on medicinal plant ingredients are important to ensure the physical and analytical characteristics of raw materials used for the production up to the standard. Additionally, product development and innovation are other key factors to succeed the herbal cosmetic industry. Thus, external collaborations with universities or research institutes will provide possible opportunities to transfer technology to herbal cosmetic industry of Sri Lanka.

## 4. Conclusion

The present study reports the first survey conducted to exploit the plants used in herbal cosmetic industry and to identify major constraints faced by herbal cosmetic manufacturers in Sri Lanka. To overcome the issues faced by herbal cosmetic manufacturers, establishing proper cultivation system and implementing strategies for quality control of raw materials have become necessary requirements of the country to be addressed in the future. Conducting ethnobotany and ethnopharmacological surveys to identify cosmetic potential of medicinal plants and partnerships with universities to transfer innovative aspects of technology for product development to industries are possible interventions for the development of herbal cosmetic industry of Sri Lanka. Furthermore, findings of the study may provide necessary information for potential growers, suppliers, manufacturers, and researchers to identify interesting cosmetic potential plants for cultivation, trading, and innovation or to use for new purposes.

## Figures and Tables

**Table 1:** Plant species used in commercialized herbal cosmetic products in Sri Lanka.

No.	Family	Scientific name	English name	Vernacular name	Plant part(s)	Availability	Source	Product category(s)	Frequency index
1	Acanthaceae	*Justicia adhatoda* L.	Malabar nut	Adathoda	Leaves	Sufficient	Local	Oral care	18.18

2	Amaranthaceae	*Alternanthera sessilis* (L.) R.Br.ex DC.	Sessile joyweed	Mugunuwenna	Whole plant	Sufficient	Local	Hair care	45.45

3	Anacardiaceae	*Mangifera indica* L.	Mango	Amba	Fruit	Sufficient	Local	Skin care	18.18

4	Apiaceae	*Daucus carota* L.	Carrot	Carrot	Root	Sufficient	Local	Skin care	27.27
5		*Centella asiatica* (L.) Urb.	Pennywort	Gotukola	Whole plant	Sufficient	Local	Skin care, hair care	54.55

6	Apocynaceae	*Plumeria obtusa* L.	Singapore graveyard flower	Araliya	Flower	Sufficient	Local	Skin care	9.09
7		*Hemidesmus indicus* (L.) R.Br.ex Schult.	Indian sarsaparilla	Iramusu	Whole plant	Sufficient	Local	Skin care	27.27

8	Arecaceae	*Cocos nucifera* L.	Coconut	Pol	Fruit	Sufficient	Local	Skin care, hair care	27.27
9		*Cocos nucifera* L.	King coconut	Thambili	Fruit	Sufficient	Local	Hair care	9.09

10	Aristolochiaceae	*Aristolochia bracteolata* Lam.	Warm killer	Sapsanda	Root	Sufficient	Local	Oral care	9.09

11	Asparagaceae	*Asparagus falcatus* L.	Sickle thorn	Hathawariya	Whole plant	Sufficient	Local	Skin care	9.09

12	Asphodelaceae	*Aloe vera* (L.) Burm.f.	Aloe	Komarika	Leaf gel	Sufficient	Local	Skin care, hair care	90.91

13	Asteraceae	*Bellis perennis* L.	Daisy	Daisy	Flower	Sufficient	Local	Skin care	9.09
14		*Genus Tagetes*	Marigold	Daspethiya	Flower	Sufficient	Local	Skin care	9.09
15		*Vernonia cinerea* (L.) Less.	Ironweed	Monarakudumbiya	Whole plant	Sufficient	Local	Skin care	9.09
16		*Genus Helianthus*	Sunflower	Suryakantha	Seed	Sufficient	Local	Skin care	18.18
17		*Saussurea costus* (Falc.) Lipsch	Costus	Suwanda Kottam	Stem	Sufficient	Local	Skin care, hair care	36.36
18		*Eclipta prostrata* (L.) L.	False daisy	Keekirindiya	Whole plant	Sufficient	Local	Hair care	54.55

19	Calophyllaceae	*Mesua ferrea* L.	Iron wood	Na	Flower stamen	Insufficient	Local	Skin care	18.18

20	Caricaceae	*Carica papaya* L.	Papaya	Gaslabu	Fruit, leaves	Sufficient	Local	Skin care	27.27

21	Celastraceae	*Kokoona zeylanica Thwaites*		Kokum	Bark	Insufficient	Local	Skin care	72.73

22	Clusiaceae	*Garcinia quaesita* Pierre	Red mango	Rath goraka	Bark, fruit rind	Sufficient	Local	Skin care, oral care	18.18

23	Combretaceae	*Terminalia chebula* Retz.	Chebulic myrobalan	Aralu	Fruit	Sufficient	Local	Oral care, hair care	45.45
24		*Terminalia bellirica* (Gaertn.) Roxb.	Beleric myrobalan	Bulu	Seed	Sufficient	Local	Hair care	9.09

25	Cucurbitaceae	*Cucumis sativus* L.	Cucumber	Pipinga	Fruit	Sufficient	Local	Skin care	36.36

26	Cyperaceae	*Cyperus rotundus* L.	Nut grass	Kalanduru	Tuber	Sufficient	Local	Skin care	18.18

27	Dilleniaceae	*Dillenia retusa* Thunb.		Godapara	Fruit	Insufficient	Local	Hair care	18.18

28	Dipterocarpaceae	*Vateria copallifera* (Retz.) Alston		Haldummala	Fruit	Sufficient	Local	Skin care	9.09

29	Elaeocarpaceae	*Elaeocarpus serratus* L.	Ceylon olive	Veralu	Leaves	Sufficient	Local	Hair care	18.18

30	Euphorbiaceae	*Ricinus communis* L.	Castor	Erandu	Leaves	Sufficient	Local	Skin care	9.09
31		*Croton aromaticus* L.		Wel keppetiya	Leaves	Sufficient	Local	Hair care	9.09

32	Fabaceae	*Cassia fistula* L.	Golden shower tree	Ehela	Leaves	Sufficient	Local	Skin care	9.09
33		*Erythrina variegata* L.	Coral tree	Erabadu	Leaves	Sufficient	Local	Skin care	9.09
34		*Senna alata* (L.) Roxb.	Emperor's candlesticks	Eththora	Leaves	Sufficient	Local	Skin care, hair care	36.36
35		*Cicer arietinum* L.	Chick pea	Kadala	Seed	Sufficient	Local	Skin care	9.09
36		*Pongamia pinnata* (L.) Pierre	Indian beech	Karanda	Bark	Sufficient	Local	Oral care	9.09
37		*Sesbania grandiflora* (L.) Pers.	Vegetable hummingbird	Kathurumurunga	Leaves	Sufficient	Local	Hair care	9.09
38		*Tephrosia purpurea* (L.) Pers.	Purple tephrosia	Kathurupila/Pila	Root	Sufficient	Local	Oral care	18.18
39		*Adenanthera pavonina* L.	Red lucky seed	Madatiya	Leaves	Sufficient	Local	Hair care	45.45
40		*Indigofera tinctoria* L.	Indigo blue	Nil–awari	Leaves	Sufficient	Local	Hair care	54.55
41		*Cassia auriculata* L.	Mature tea tree	Ranwara	Flower	Sufficient	Local	Skin care	9.09
42		*Pterocarpus santalinus* L.f.	Red sandalwood	Rath Handun	Heartwood	Sufficient	Import	Skin care, hair care	18.18
43		*Trigonella corniculata* Sibth. & Sm.	Sickle-fruit fenugreek	Siyakka	Seed	Sufficient	Local	Hair care	18.18
44		*Tamarindus indica* L.	Tamarind	Siyambala	Seed	Sufficient	Local	Skin care	18.18
45		*Trigonella foenum-graecum* L.	Fenugreek	Uluhal	Seed	Sufficient	Local	Hair care	36.36
46		*Phaseolus mungo* L.	Black gram	Undu	Seed	Sufficient	Local	Skin care	9.09
47		*Glycyrrhiza glabra* L.	Liqourice	Walmee	Root	Sufficient	Import	Skin care, hair care, oral care	54.55

48	Lamiaceae	*Ocimum tenuiflorum* L.	Tulsi	Heen Maduruthala	Leaves	Sufficient	Local	Skin care	18.18
49		*Plectranthus amboinicus* (Lour.) Spreng.	Country borage	Kapparawalliya	Leaves	Sufficient	Local	Skin care	9.09

50	Lauraceae	*Persea americana* Mill.	Avocado pear	Aligata pera	Fruit	Sufficient	Local	Skin care	18.18
51		*Cinnamomum verum* J.Presl	Cinnamom	Kurundu	Bark	Sufficient	Local	Skin care, hair care, oral care	36.36

52	Loganiaceae	*Strychnos potatorum* L.f.	Clearing-nut tree	Ingini	Seed	Sufficient	Local	Skin care	9.09

53	Lythraceae	*Punica granatum* L.	Pomegranate	Delum	Leaves, fruit rind	Sufficient	Local	Skin care	18.18
54		*Lawsonia inermis* L.	Henna	Marathondi	Leaves	Sufficient	Local	Hair care	18.18

55	Malvaceae	*Thespesia populnea* (L) Sol.ex Correa	Portia tree	Gansooriya	Leaves, bark	Sufficient	Local	Skin care	18.18
56		*Theobroma cacao* L.	Cocoa	Kokova	Seed	Sufficient	Local	Skin care	9.09
57		*Hibiscus rosa-sinensis* L.	China rose	Pokuru wada	Flower	Sufficient	Local	Hair care	27.27
58		*Abelmoschus moschatus* Medik.	Musk mallow	Kapu kinissa	Seed	Sufficient	Local	Skin care	9.09
59		*Sida cordata* (Burm.f.) Borss.Waalk.	Heart leaf sida	Wel babila	Leaves, stem	Sufficient	Local	Skin care, hair care	9.09

60	Melastomataceae	*Osbeckia octandra* DC.		Heenbowitiya	Flower	Sufficient	Local	Skin care	9.09

61	Meliaceae	*Azadirachta indica* A.Juss.	Neem	Kohomba	Bark, leaves	Sufficient	Local	Skin care, hair care	72.73

62	Menispermaceae	*Tinospora cordifolia* (Willd.) Miers	Heart leaved mooseed	Rasakinda	Stem	Sufficient	Local	Skin care	9.09
63		*Coscinium fenestratum* (Goetgh.) Colebr	Calumba wood	Weni wel	Stem	Insufficient	Local	Skin care	90.91

64	Molluginaceae	*Mollugo cerviana* (L.) Ser.	Threadstem carpetweed	Pathpadagam	Whole plant	Sufficient	Local	Skin care	9.09

65	Moraceae	*Ficus religiosa* L.	Sacred fig	Bo	Bark	Sufficient	Local	Skin care	9.09
66		*Morus alba* L.	Silkworm mulberry	Mulberry	Fruit	Sufficient	Local	Skin care	9.09
67		*Ficus benghalensis* L.	Banyan tree	Nuga	Bark	Sufficient	Local	Skin care	9.09
68		*Ficus racemosa* L.	Cluster fig tree	Attikka	Bark	Sufficient	Local	Skin care	9.09

69	Moringaceae	*Moringa oleifera* Lam.	Drumstick tree	Murunga	Seed, leaves	Sufficient	Local	Skin care	18.18

70	Myristicaceae	*Myristica fragrans* Houtt.	Nutmeg	Sadhikka	Seed	Sufficient	Local	Hair care	9.09
71	Myrtaceae	*Caryophyllus aromaticus* L.	Clove	Karabu	Flower buds	Sufficient	Local	Skin care, oral care	54.55
72		*Eucalyptus globulus* Labill.	Tasmanian bluegum	Karupantine	Leaves	Sufficient	Local	Oral care	9.09
73		*Melaleuca leucadendra* (L.) L.	Cajuput tree	Lothsumbulu	Bark	Insufficient	Local	Skin care	18.18
74		*Psidium guajava* L.	Guava	Pera	Leaves	Sufficient	Local	Hair care	18.18

75	Nelumbonaceae	*Nelumbo nucifera* Gaertn.	Lotus	Nelum	Flower stamen	Sufficient	Local	Skin care	27.27

76	Nymphaeaceae	*Nymphaea nouchali* Burm.f	Blue water lily	Nil-manel	Flower stamen	Sufficient	Local	Skin care	9.09

77	Oleaceae	*Olea europaea* L.	Olive	Olive	Fruit	Sufficient	Import	Skin care, hair care	27.27
78		*Jasminum grandiflorum* L.	Jasmine	Saman Pichcha	Flower	Sufficient	Local	Skin care, hair care	36.36

79	Pandanaceae	*Pandanus tectorius* Parkinson ex Du Roi	Tahitian screwpine	Watakeyya	Prop roots	Sufficient	Local	Hair care	9.09

80	Passifloraceae	*Passiflora edulis* Sims	Passion	Wel dodam	Fruit	Sufficient	Local	Skin care	18.18

81	Pedaliaceae	*Sesamum indicum* L.	Sesame	Thel - thala	Seed	Sufficient	Local	Skin care, hair care	45.45

82	Phyllanthaceae	*Phyllanthus emblica* L.	Emblic myrobalan	Nelli	Fruit	Sufficient	Local	Skin care, hair care	63.64

83	Pinaceae	*Cedrus deodara* (Roxb. ex D.Don) G.Don	Himalayan cedar	Dewadara	Heartwood	Sufficient	Import	Skin care	9.09

84	Piperaceae	*Piper nigrum* L.	Black pepper	Gammiris	Seed	Sufficient	Local	Oral care	18.18

85	Plantaginaceae	*Bacopa monnieri* (L.) Wettst.	Water hyssop	Lunuwila	Whole plant	Insufficient	Local	Skin care, hair care	27.27

86	Poaceae	*Cymbopogon nardus* (L.) Rendle	Citronella grass	Heen pengiri	Whole plant	Sufficient	Local	Skin care	9.09
87		*Oryza sativa* L.	Red rice	Rathu hal	Bran	Sufficient	Local	Skin care	9.09
88		*Cymbopogon citratus* (DC.) Stapf	Lemon grass	Sera	Whole plant	Sufficient	Local	Skin care	18.18
89		*Saccharum officinarum* L.	Sugarcane	Uk	Stem	Sufficient	Local	Skin care	18.18
90		*Vetiveria zizanioides* (L.) Nash	Khas-khas	Savandara	Root	Sufficient	Local	Hair care	36.36

91	Pontederiaceae	*Monochoria vaginalis* (Burm.f.) C.Presl		Diyahabarala	Root	Sufficient	Local	Hair care	9.09
92	Rosaceae	*Prunus armeniaca* L.	Apricot	Apricot	Seed	Sufficient	Import	Skin care	18.18
93		*Rosa alba* L.	Rose	Rosa	Flower	Sufficient	Local	Skin care	27.27
94		*Fragaria × ananassa* Duchesne	Strawberry	Strawberry	Fruit	Sufficient	Import	Skin care	18.18

95	Rubiaceae	*Coffea arabica* L.	Coffee	Kopi	Seed	Sufficient	Local	Skin care	9.09
96		*Ixora coccinea* L.	Flame of the woods	Rath mal	Flower	Sufficient	Local	Skin care	9.09
97		*Rubia cordifolia* L.	Indian maddar	Valmadata	Root	Sufficient	Local	Skin care	27.27

98	Rutaceae	*Acronychia pedunculata* (L.) Miq.	Claw flowered laure	Ankenda	Leaves	Sufficient	Local	Hair care	18.18
99		*Ruta chalepensis* L.	Garden rue	Aruda	Leaves	Sufficient	Local	Skin care, hair care	18.18
100		*Aegle marmelos* (L.) Corrêa	Bael fruit tree	Beli	Fruit	Sufficient	Local	Skin care	9.09
101		*Citrus aurantiifolia* (Christm.) Swingle	True lime	Dehi	Fruit, fruit rind	Sufficient	Local	Skin care, hair care	54.55
102		*Citrus × sinensis*	Orange	Dodam	Fruit, fruit rind	Sufficient	Local	Skin care	27.27
103		*Citrus limon* (L.) Osbeck	Lemon	Lemon	Fruit, fruit rind	Sufficient	Local	Skin care, hair care	27.27
104		*Citrus sinensis* (L.) Osbeck	Sweet orange	Pani dodam	Fruit, fruit rind	Sufficient	Local	Skin care	9.09

105	Santalaceae	*Santalum album* L.	Sandalwood	Suduhandun	Heartwood	Insufficient	Import	Skin care	90.91

106	Sapindaceae	*Sapindus mukorossi* Gaertn.	Soap nut	Gas penela	Fruit	Sufficient	Local	Hair care	9.09
107		*Schleichera oleosa* (Lour.) Merr.	Ceylon oak	Kon	Seed	Sufficient	Local	Hair care	18.18

108	Sapotaceae	*Mimusops elengi* L.	Bullet wood tree	Munamal	Bark	Sufficient	Local	Oral care	36.36

109	Solanaceae	*Withania somnifera* (L.) Dunal	Indian ginseng	Amukkara	Root	Sufficient	Import	Skin care	27.27

110	Theaceae	*Camellia sinensis* L. Kuntze	Tea	Thae	Tender leaves	Sufficient	Local	Skin care	18.18

111	Zingiberaceae	*Curcuma zedoria* (Christm.) Roscoe	Zedoary	Harankaha	Rhizome	Sufficient	Local	Skin care	9.09
112		*Zingiber officinale* Roscoe	Ginger	Inguru	Rhizome	Sufficient	Local	Skin care, oral care	18.18
113		*Curcuma longa* L.	Turmeric	Kaha	Rhizome	Sufficient	Local	Skin care	54.55
114		*Curcuma aromatica* Salisb.	Wild turmeric	Kasthuri kaha	Rhizome	Sufficient	Local	Skin care	54.55
115		*Alpinia malaccensis* (Burm.f.) Roscoe		Rankihiriya	Flower bud	Sufficient	Local	Oral care	9.09

## Data Availability

The data used to support the findings of this study are included within the article.

## Supplementary Materials

Supplementary Materials Supplementary Material 1: the questionnaire which was used to collect the information on plants/plant materials used in herbal cosmetic industry of Sri Lanka and some demographic information of the herbal cosmetic manufacturers.
