# Supplementary material for: Emerging Herbal Cosmetic Production in Sri Lanka: Identifying Possible Interventions for the Development of the Herbal Cosmetic Industry
Source: Scientifica (Cairo). 2021 Mar 10;2021:6662404. doi: 10.1155/2021/6662404 (PMC7984923; doi:10.1155/2021/6662404)
Supplement: Supplementary Materials — Supplementary Material 1: the questionnaire which was used to collect the information on plants/plant materials used in herbal cosmetic industry of Sri Lanka and some demographic information of the herbal cosmetic manufacturers. [file 6662404.f1.docx]

**Supplementary material 1**

**Questionnaire form for identifying of plants used in herbal cosmetic industry of Sri Lanka**

Section 01

1. Name: …………………………………………………………………………………
2. Organization: ………………………………………………………………………….
3. Experience of the profession (years): …………………………………………………
4. Registration number: ………………………………………………………………….
5. Address: ……………………………………………………………………………….
6. Province/ District:

| Uva | Badulla |  |
| --- | --- | --- |
|  | Monaragala |  |

| Northern | Jaffna |  |
| --- | --- | --- |
|  | Kilinochchi |  |
|  | Mannar |  |
|  | Mullaitivu |  |
|  | Vavuniya |  |

| Sabaragamuwa | Kegalle |  |
| --- | --- | --- |
|  | Ratnapura |  |

| Eastern | Trincomalee |  |
| --- | --- | --- |
|  | Batticaloa |  |
|  | Ampara |  |

| North Western | Puttalam |  |
| --- | --- | --- |
|  | Kurunegala |  |

| North Central | Anuradhapura |  |
| --- | --- | --- |
|  | Polonnaruwa |  |

| Southern | Hambantota |  |
| --- | --- | --- |
|  | Matara |  |
|  | Galle |  |

| Western | Gampaha |  |
| --- | --- | --- |
|  | Colombo |  |
|  | Kalutara |  |

| Central | Matale |  |
| --- | --- | --- |
|  | Kandy |  |
|  | Nuwaraeliya |  |

Section 02

Please mention about your manufacturing product/s with brand names.

| **Skin Care** | **Hair Care** | **Dental Care** |
| --- | --- | --- |
|  |  |  |
|  |  |  |
|  |  |  |
|  |  |  |
|  |  |  |
|  |  |  |
|  |  |  |
|  |  |  |

What kind of medicinal plants are required for your production/s.

| **Medicinal Plant** | **Plant part/s** | **Volume (kg/year)** | **Method of receiving raw materials (Local/Import)** | **Availability of raw materials**  **(Sufficient/ non-sufficient/ partially receive)** |
| --- | --- | --- | --- | --- |
|  |  |  |  |  |
|  |  |  |  |  |
|  |  |  |  |  |
|  |  |  |  |  |
|  |  |  |  |  |
|  |  |  |  |  |
|  |  |  |  |  |
|  |  |  |  |  |
|  |  |  |  |  |
|  |  |  |  |  |
|  |  |  |  |  |
|  |  |  |  |  |
|  |  |  |  |  |
|  |  |  |  |  |
|  |  |  |  |  |
|  |  |  |  |  |
|  |  |  |  |  |
|  |  |  |  |  |

What are the main difficulties that you face in getting medicinal plants for your production/s?

…………………………………………………………………………………………………………………………………………………………………………………………………………………………………………………………………………………………………………………………………………………………………………………………………………………………………………………………………………………………………………………………………………………………………………………………………………………………………………………………………………………………………………………………………………………………………………

Add your suggestions for improving the research based on the medicinal plants in cosmetics (future potential research areas).

…………………………………………………………………………………………………………………………………………………………………………………………………………………………………………………………………………………………………………………………………………………………………………………………………………………………………………………………………………………………………………………………………………………………………………………………………………………………………………………………………………………………………………………………………………………………………………
